# Banding cytogenetic analysis in pediatric patients with acute lymphoblastic leukemia (ALL) in a Brazilian population

**DOI:** 10.1186/1755-8166-6-37

**Published:** 2013-09-11

**Authors:** Erica Aires Gil, Tirzah Braz Petta Lajus, Taissa Maria Oliveira de Moura, Juliana Mendonça Freire, Andréa Luciana Araújo da Fernandes, Gioconda Dias Rodrigues Leão, Edlene Melo Reis do Nascimento, Gabriela Vasconcelos Andrade de Alves, Geraldo Barroso Cavalcanti Júnior

**Affiliations:** 1 Departamento de Análises Clínicas e Toxicológicas/ Laboratório de Imunologia, Universidade Federal do Rio Grande do Norte/, Rua Gen, Gustavo Cordeiro De Farias S/N. Petrópolis, CEP 59010–180, Natal, RN, Brazil; 2 Liga contra o Câncer. Departamento de Pesquisa Translacional, Av. Miguel Castro 1355. Dix-Sept Rosado, CEP 59062–000, Natal, RN, Brazil

**Keywords:** (3 a 6), Epidemiology, Acute lymphoblastic leukemia, Childhood, Banding cytogenetic analysis

## Abstract

**Background:**

Cytogenetic studies in Brazilian population about childhood acute lymphoblastic leukemia (ALL), the most common childhood malignancy, are scarce. Moreover, Brazilian race is very heterogeneous and is made by the confluence of people of several different origins, from the original Native Brazilians, with the influx of Portuguese colonizers, Black African slaves, and recent European, Arab and Japanese immigration. The purpose of this prospective, multicentric study was to assess the sociodemographic, clinic and cytogenetic characteristics of the children treated for ALL in the Northeast region of Brazil.

**Results:**

This study includes thirty patients between 4 months and 17 years old treated for ALL from January 1st, 2009 to November 30th, 2010. Cytogenetic analysis showed that in nineteen out of thirty patients (64%) presented some chromosome abnormalities, in which 53% corresponds to numerical abnormalities, 21% structural and numerical abnormalities, and 26% only structural changes. Moreover, seven patients presented complexes karyotype not yet described in the literature. Taken together these results show the importance of the cytogenetic analysis in ALL pediatric patients and illustrates that the studied population presented unexpected complexes karyotypes which were correlated to poor outcome.

**Conclusion:**

The results demonstrate the importance of banding cytogenetics for ALL diagnosis despite the use of most modern techniques such as FISH and aCGH, and provide reliable insight into the ALL in Brazil.

## Background

Acute lymphoblastic leukemia (ALL) comprises a group of neoplasms characterized by clonal proliferation and accumulation in the blast, hematopoietic immature cells in the bone marrow. ALL is a disease characterized by abnormal clonal proliferation of lymphoid precursors which lose their ability to differentiate. It is the most common malignancy in childhood, accounting for 80% of all leukemia. Although it can occur at any age, its incidence is highest among children from 2 to 5 years at a rate of about 70%, decreasing among adolescents and young adults. The most useful prognostic indicators in ALL are age, white blood cell count (WBC) and immunologic markers. Cytogenetic analysis is of fundamental importance in the diagnosis, therapy, in monitoring post-transplant bone marrow and to assess prognosis. It is now possible to correlate the karyotype with other recognized prognostic factors and to show that cytogenetic studies can increase the precision of other clinical prognostic features. Unfortunately, banding cytogenetic analysis of leukemia is technically demanding, especially in the case of ALL in infants. Clonal abnormalities are difficult to identify because of frequent failure of standard chromosome-preparing protocols. Moreover, clonal selection leads to a high proportion of normal metaphases, masking abnormal cells when a limited number of metaphases are analyzed [1]. Therefore, the chromosomal classification of ALL is often incomplete, and it is not clear whether the abnormalities detected in a few analyzable metaphases are representative of the whole leukemic clone [2,3].

Banding cytogenetic studies have identified numerical and structural chromosomal abnormalities related to the disease’s pathophysiologic characteristics. Frequency of these abnormalities varies between pediatric and adult patients. In children, pseudodiploidy is the most frequent (40%), followed by hyperdiploidy with 51–65 chromosomes (27%), hyperdiploidy with 47–50 chromosomes (15%), hypodiploidy (6%), near-haploidy (1%), and the tetraploids (1–2%) [4,5]. It has also been demonstrated that the frequency with which chromosomal abnormalities are observed varies among populations. In the Hindu population, hyperdiploidy is infrequent (15%), in contrast with the high frequency of hypodiploidy (38.4%). This behavior has been attributed to unknown ethnic differences and geographic factors [6,7].

We aimed to study the cytogenetic profile in pediatric patients in several health institutions in the Brazilian Northeast region, which is a very heterogeneous race and no data on this feature is available in the literature.

## Aim

To determine the cytogenetics profile of patients with ALL during the period from January 1st, 2009 to November 30th, 2010 in the Northeast of Brazil.

## Results

### Patients characteristics

The sample comprised 30 patients diagnosed for ALL between one and ten years old. The clinical characteristics of the ALL patient populations are provided in Table 1. Thirty seven percent (37%) were in the range between two and five years old, with a mean age of 7,5 years old. Regarding the sex, the male:female ratio was 1,3:1. The complexity of racial classifications in Brazil reflects the extent of miscegenation in Brazilian society, more people would report themselves as white and *pardo*, which is a mixed race of white and black people found only in Brazil (47.7% and 42.4% of the population as of 2010, respectively). For this reason we have separated the studied population by skin color. Skin color was predominantly white (50%) followed by *pardo* (33%) and black (17%).

**Table 1:** **An unusual T**-**cell childhood acute lymphoblastic leukemia harboring a yet unreported near**-**tetraploid karyotype**

**N**	**Sex**	**Age (y)**	**Skin color**	**BCC (g/dL)**	**Leucometry values (ul)**	**Platelets values (ul)**
1	F	4	White	7,3	20200	32000
2	F	7	White	12	11000	44000
3	M	5	Pardo	5,9	34700	33000
4	M	16	White	13	4600	63000
5	F	6	White	7,5	3100	32000
6	F	4	Black	8	4300	283000
7	M	3	Black	9	5800	336000
8	F	5	White	10,2	7800	165000
9	M	12	White	14	4400	249000
10	M	16	Black	8	1500	12000
11	M	17	Pardo	15	2800	19000
12	F	15	White	8	3000	52000
13	M	8	Pardo	11,3	3200	44900
14	F	0,4	Black	7,8	96600	65000
15	M	3	Pardo	9,2	21900	55000
16	F	3	Black	7,3	7900	26000
17	M	5	Pardo	6,8	35900	54000
18	M	2	White	5,7	6300	7000
19	M	3	White	8,4	50900	37000
20	F	1	Pardo	5,4	45700	26000
21	M	17	Pardo	7,1	60000	12000
22	F	7	Pardo	9,4	33200	83000
23	F	3	White	7,5	5600	45000
24	F	6	White	9,4	25000	38000
25	F	17	White	11,3	6500	18000
26	M	2	White	8,5	7400	45000
27	M	0,4	Pardo	10	13500	60000
28	M	6	White	7,8	2100	42000
29	M	5	White	6,9	5600	77000
30	M	16	Pardo	11,5	5300	160000

According to medical records, the most common finding of physical examination was hepatomegaly (73%), splenomegaly (70%) and lymphadenopathy (37%). The samples (bone marrow and peripheral blood) were collected previous treatment in 83% of patients, whereas 17% were submitted to collection after treatment has been initialized. Seventy three percent (73%) of the patients’ hemoglobin levels were less than 10 g/dL; 60% of patients showed white blood cell (WBC) counts below 10.000/μL and 40% above this value. Considering the population who showed WBC above 10.000/μL, 10% were above 50.000/μL. Thrombocytopenia was observed in 83% of cases (Table 1).

### Cytogenetics studies

Cytogenetic studies using G-banding techniques were performed in 30 samples from ALL patients, where nineteen (64%) presented chromosomal alterations in the bone marrow (BM) and peripheral blood (PB), seven patients (23%) had normal karyotype and in four patients (13%) it was not possible to analyze due to the absence of cell growth (MI - mitotic index zero). Immunophenotype analysis showed that 93% of patients had B-lineage ALL and 7% T- lineage ALL. Among the B-lineage ALL, the majority of cases (83%) were classified as pre-B (Table 2). The small percentage of patients with T-ALL may be associated with the proportion of adolescent patients in this sample which were over 10 years.

**Table 2:** **Results from G**-**banding technique related to imunophenotype of each patient** (**1**–**30**)

**N**	**Karyotype**	**Cell type**	**Classification of cytogenetic alteration**	**Ploidy**
**1**	42 ~ 45,XX, -3[3], -13[3], -20[4][cp7] /46,XX [13]	ALL pre-B (L1)	Numerical	Hyperdiploidy
**3**	45 ~ 56,XY, +4[3], +8[4], +11[3], +18[4], +20[3], +21[4][cp20] / 46, XY [05]	ALL pre-B (L1)	Numerical	Hyperdiploidy
**4**	46,XY [20]	ALL pre-B (L3)	Normal	-
**5**	46, XX [13]	ALL pre-B (L1)	Normal	-
**6**	46, XX [23]	ALL pre-B (L1)	Normal	-
**7**	46,XY, del(4)(p14?) [09] / 46,XY [11]	ALL pre-B (L1)	Structural	Pseudodiploidy
**8**	49 ~ 58,XX, +5[4], +9[3], +11[3], +15[4], +21[4], +22[3] [cp9] / 46,XX [02]	ALL pre-B (L1)	Numerical	Hyperdiploidy
**9**	46,XY [23]	ALL pre-B (L1)	Normal	-
**11**	48, XY, t (1;3) (q32;q27), +16, + mar[09] / 46, XY [14]	ALL pre-B (L2)	Numerical + structural	Hyperdiploidy+add
**12**	49 ~ 87, XX , +1[5], +3[4], +5[5], +7[5], +10[4], +12[6], +13[04], add(14)(q32?), add(16)(p13.3), +18[6], +19[4], +20[3], +21[6], +22[5][cp30]	ALL Pre-B (L1)	Numerical + structural	Hyperdiploidy+add
**13**	49~54, XY, +3[3], +5[4], +8[4], +10[3], +17[4], +21[4][cp11] / 46,XY [04]	ALL pre-B (L3)	Numerical	Hyperdiploidy
**14**	46,XX,del(11)(q23) [17]	ALL pre-B (L1)	Structural	Pseudodiploidy
**15**	41~45,XY, -5[3], -7[3], -21[4][cp13] / 46,XY[07]	ALL pre-B (L1)	Numerical	Hypodiploidy
**16**	50~61,XX, +2[3], +4[4], +10[4], +12[3],+16[4], +19[5], +21[4][cp7] / 46,XX[05]	ALL pre-B (L1)	Numerical	Hyperdiploidy
**17**	48~60,XY, +3[4], +5[5], +7[4], +13[4], +17[3], +20[4], +21[4][cp22] / 46,XY[05]	ALL pre-B (L1)	Numerical	Hyperdiploidy
**18**	49~58,XY, +1[4], +6[4], +7[5], +22[3][cp9] / 46,XY[11]	ALL pre-B (L1)	Numerical	Hyperdiploidy
**19**	46,XY, dup(1)(q31q44) [12] / 46,XY, dup(13)(q13q34) [05] / 46,XY, der(21) t(7;21) (q21q36;q22.3) [04] / 46,XY [04]	ALL – B (L3)	Structural	Pseudodiploidy
**20**	46, XX, -7, + der 7 t(7;?)(q31;?) [30]	ALL pre-B (L1)	Numerical + structural	Pseudodiploidy
**23**	53~62,XX, +5[4], +8[4], +12[3], +16[4], +18[3], +20[4], +21[4][cp7]/46,XX[15]	ALL-B (L2)	Numerical	Hyperdiploidy
**24**	46,XX[19]	ALL pre-B (L3)	Normal	-
**25**	46,XX,t(9;22)(q34;q11), add(14)(q32) [20]	ALL pre-B (L1)	Structural	Pseudodiploidy
**26**	47,XY,i(7)(q10), +21c[22] /48,idem, +mar[4] / 48, XY, i(7)(q10), +8, +21c [03]	ALL pre-B (L1)	Numerical + structural	Hyperdiploidy+add
**27**	46,XY [20]	ALL – T (L2)	Normal	-
**28**	38~45,XX, t(9;14), (q22?;q32?), del(20)(q?) [21]	ALL pré-B (L2)	Numerical + strutural	Hypodiploidy+add
**29**	46,XY [20]	ALL pré-B	Normal	-
**30**	36~45,XY, -4[3], -7[4], -9[3], -11[3], -22[4] [cp23] / 46,XY [03]	ALL pré-B	Numerical	Hypodiploidy

Legend: Samples 2, 10, 21 and 22 had null Mitotic Index (MI=0). Complex karyotypes are highlighted in bold character in the table (N=7). L1: acute lymphoblastic leukemia type 1; L2: acute lymphoblastic leukemia type 2; L3: acute lymphoblastic leukemia type 3. inv - inversion; mar – marker chromosome; t – translocation; i – isochromosome; add – additional chromosome; dup – duplication; der – derivative chromosome; Ph+ - Philadelphia chromosome; del – deletion; p – short arm of the chromosome; q – long arm of the chromosome, [ ] cells number analyzed.

Complex karyotype are highlighted in the table (N=7).

Considering the nineteen patients who presented some type of chromosomal abnormality, 53% were numerical abnormalities, 21%, were structural and numerical abnormalities and 26% of the patients presented only structural changes. In fourteen patients who presented numeric chromosomal abnormalities and numeric alteration with additional structural change, hyperdiploidy was detected in eight patients (57%); hyperdiploidy with additional change in three patients (22%); hypodiploidy in two patients (14%), and finally, hypodiploidy with additional changes in one patient (7%) (Table 2). Unusual or novel cytogenetic abnormalities were found in 7 patients (23%). The karyotypes included in these cases presented hyper or hypodiploidy with additional modifications, in addition to the presence of cells with chromosomal markers (Table 2). Patient 26 is a child with Down syndrome (DS) and acute lymphoblastic leukemia (ALL) and is classified as complex karyotype for presenting three different clones.

## Discussion

Leukemia is the most common malignant neoplasms in childhood, accounting for approximately 33% of all malignancies in children under 14 years. Leukemia incidence affects the 0–14 years old population with a frequency of 1/25.000 individuals/ year, and the risk of developing the disease within the first 10 years is 1/2.880. After the discovery by Casperson [8] and Seabright [9] of banding techniques, cytogenetic studies showed that about 50% of chromosomal abnormalities could be found in acute leukemia. Several prospective studies using banding techniques, have shown that the incidence of chromosomal abnormalities varies between 55-94%, and found that some changes are specific to particular cell subtype characterized by immunophenotyping [10,11].

Considering our results, where fifteen patients presented numerical chromosomal anomalies and numerical structural change, the hyperdiploidy was observed in ten patients (67%), which showed 54% only hyperdiploidy and 13% with hyperdiploidy with additional changes, whereas hypodiploidy was found in 33% of patients. Of these, 13% had some structural change. The additional structural changes found were translocation, addition, duplication and the presence of chromosome marker. Pérez-Vera et al. [12] found that 22% patients presented normal karyotype and 74% had some cytogenetic abnormalities. Pui et al. [13] evaluated children diagnosed with ALL and found hyperdiploidy in 26% of the patients. In 62% of these cases were also detected chromosomal structural changes.

According to the literature, it is considered complex karyotypes more than one chromosomal abnormality and/or an alteration which has not yet been described. We observed seven novel complex karyotypes, among these, a translocation between chromosomes 9 and 22 (Ph+) with additional chromosome 14, duplication of chromosomes 1q and 13q, and a derivative of chromosome 21 translocated to chromosome 7, forming a derivative of chromosome 7. It is important to highlight that these complex karyotypes are considered unfavorable outcome. One possible reason could be due to loss, gain or exchange of genes that can promote often resistant to treatment. The presence of the Philadelphia chromosome (Ph+) or 9:22 translocation is usually found in about 3-5% of the pediatric ALL, and is a modification of poor prognosis [14]. It was observed the presence of a marker chromosome in three analyzed karyotype. The marker chromosomes are complex rearrangements, usually consisting of centromeric heterochromatin, but may contain up to part of a chromosome gene. It is practically impossible to establish a precise relationship between the presence of this type of chromosome and the phenotype of the patient. Thus, the characterization of each chromosome marker becomes imperative for this correlation [15].

Among the complex karyotypes it has been found a Down syndrome (DS) presenting three clones cell. According to this finding, Silva et al. [16] found a karyotype of a DS patient involving four clones. In the literature, the relative risk of acute leukemia in the first 5 years has been estimated to be 56 times that of non-DS individuals with an equal frequency of acute myeloid leukemia (AML) or acute lymphoblastic leukemia (ALL) [17].

This study could not be supplemented by molecular approaches due to limitations in amount of patient material and technical limitations as present in many regions of the world. However, banding cytogenetics is the basics for all further studies, especially in rare malignancies like the here reported childhood leukemias.

Even though it is difficult to assume the outcome of these patients, patients with complex cytogenetic changes were classified as poor prognosis. The prognosis was considered favorable in patients with hyperdiploidy or with normal karyotype, and the age between one and six years and/or leukocyte count close to normal. The intermediate prognosis has been established for patients with karyotypes who had only one structural chromosomal alteration, aged between one and six years old, leukocyte count above 20,000/μl and response to treatment assessed by the presence of blasts in the PB. The poor prognosis group presented complex karyotype, are age over 10 years old and have the presence of blasts above 25% in PB.

Taken together these results show the importance of the cytogenetic analysis in ALL pediatric patients and illustrates that the studied population presented unexpected complexes karyotypes which were correlated to poor outcome.

## Material and methods

### Study design

Patients included were issued from 5 different hospitals of the state of Rio Grande do Norte: Hospital Infantil Varela Santiago, Liga Norte-Riograndense Contra o Câncer, Centro de Oncologia e Hematologia de Mossoró, Clínica DNA Center during the period comprised between January 1st, 2009 and November 30, 2010. The inclusion criteria were cytological and immunohistochemical diagnosis for ALL; patients aged 0–18 years; availability of biological material for cytogenetic analysis; laboratory data, such as leukocyte counts, percentage of blast cells, count platelets and hemoglobin; clinical data: presence or absence of visceromegalies.

### Samples processing

Samples of bone marrow and peripheral blood of thirty patients diagnosed with ALL of both sexes, aged from 4 months to 17 years old were collected. Cytogenetic analysis on bone marrow cells and / or peripheral blood was performed in the clinical laboratory of DNA Center. The cytochemical diagnosis, morphological and immunohistochemical were provided by company Hemonorte Hematology (Natal/RN). For cytogenetic analysis, 5 ml of each bone marrow samples was collected in a syringe containing sodium heparin. 2–5 ml of bone marrow were collected in a tube containing 0,1 ml of Liquemine® Roche®. For peripheral blood, 5 ml were collected in a tube containing heparin. Samples were processed by centrifugation (2.000 rpm).

### Cell culture

The biological material was incubated overnight in sterile conditions, in media containing 8% RPMI 1640 supplemented with 20% serum fetal bovine (FBS) and glutamine (GIBCO Invitrogen®). The mitotic interruption was proceed by adding 16ug/mL colchicine (GIBCO) and incubated for 1 hour at 37°C. Cells were submitted to hypotonic treatment with a solution of 0.075 M potassium chloride for 20 minutes and the material were fixed with 3:1 (v/v) methanol: acetic acid. Cells were spread in the slides and dried on a heating plate.

### GTG-banding cytogenetics

Karyotyping was performed using standard techniques, and the results were reported according to the International System for Human Cytogenetic Nomenclature guidelines. The slides were incubated in Sorensen’s buffer for 45 min and then stained with Wright’s stain buffer for 2 minutes. The slides were analyzed in an optical microscope (Nikon-E200). A complex aberrant karyotype was defined as three or more independent cytogenetic abnormalities in at least two bone marrow cells.

### Data collection

Data were collected and entered in Microsoft Excel® spreadsheet, and later, there was a descriptive analysis using SPSS Statistics 17.0, mean, median and standard deviation of patient characteristics (age, sex, color) clinical data (lymphadenopathy, hepatomegaly and splenomogaly), chromosomal abnormalities present in the samples, prognosis of abnormalities found and, finally, the parameters hematology (hemoglobin, leukocytes and platelets).

## Ethics consent

This study was approved in the Ethics Committee of The Hospital Universitário Onofre Lopes (Universidade Federal do Rio Grande do Norte) under the registration number: 083/07. The written informed consent number obtained from the patient’s parents for publication of this case report is retained with the same Ethics Committee.

## Abbreviations

ALL: Acute lymphoblastic leukemia; AML: Acute myeloid leukemia; BM: Bone marrow; DS: Down syndrome; PB: Peripheral blood; WBC: White blood cell.

## Competing interests

Erica Gil holds a CNPq grant and TBPL holds a CAPES grant. The remaining authors declare that they have no competing interests.

## Authors’ contributions

EAG made all experiments and wrote the article, TBPL, TMOM, JMF, JMF, ALACF, GDRL helped to write the manuscript and performed data collection from medical records, EMRN helped to collect and process patient samples and GBCJ helped to conduct the study as supervisor. All authors read and approved the final manuscript.

## Authors’ information

This study was conducted by Erica Gil, MPharm, in order to obtain Master Degree at the Pharmaceutical Program at the Universidade Federal do Rio Grande do Norte.
